# Machine learning applications to classify and monitor medication adherence in patients with type 2 diabetes in Ethiopia

**DOI:** 10.3389/fendo.2025.1486350

**Published:** 2025-03-20

**Authors:** Ewunate Assaye Kassaw, Ashenafi Kibret Sendekie, Bekele Mulat Enyew, Biruk Beletew Abate

**Affiliations:** ^1^ Department of Biomedical Engineering, Institute of Technology, University of Gondar, Gondar, Ethiopia; ^2^ Center for Biomedical Engineering, Indian Institute of Technology, Delhi, New Delhi, India; ^3^ Department of Clinical Pharmacy, School of Pharmacy, College of Medicine and Health Sciences, University of Gondar, Gondar, Ethiopia; ^4^ Curtin Medical School, Faculty of Health Sciences, Curtin University, Bentley, WA, Australia; ^5^ Department of Information Technology, College of Informatics, University of Gondar, Gondar, Ethiopia; ^6^ College of Health Science, Woldia University, Woldia, Ethiopia; ^7^ School of Population Health, Curtin University, Bentley, WA, Australia

**Keywords:** medication adherence, diabetes, machine learning, classification, prediction

## Abstract

**Background:**

Medication adherence plays a crucial role in determining the health outcomes of patients, particularly those with chronic conditions like type 2 diabetes. Despite its significance, there is limited evidence regarding the use of machine learning (ML) algorithms to predict medication adherence within the Ethiopian population. The primary objective of this study was to develop and evaluate ML models designed to classify and monitor medication adherence levels among patients with type 2 diabetes in Ethiopia, to improve patient care and health outcomes.

**Methods:**

Using a random sampling technique in a cross-sectional study, we obtained data from 403 patients with type 2 diabetes at the University of Gondar Comprehensive Specialized Hospital (UoGCSH), excluding 13 subjects who were unable to respond and 6 with incomplete data from an initial cohort of 422. Medication adherence was assessed using the General Medication Adherence Scale (GMAS), an eleven-item Likert scale questionnaire. The responses served as features to train and test machine learning (ML) models. To address data imbalance, the Synthetic Minority Over-sampling Technique (SMOTE) was applied. The dataset was split using stratified K-fold cross-validation to preserve the distribution of adherence levels. Eight widely used ML algorithms were employed to develop the models, and their performance was evaluated using metrics such as accuracy, precision, recall, and F1 score. The best-performing model was subsequently deployed for further analysis.

**Results:**

Out of 422 enrolled patients, 403 data samples were collected, with 11 features extracted from each respondent. To mitigate potential class imbalance, the dataset was increased to 620 samples using the Synthetic Minority Over-sampling Technique (SMOTE). Machine learning models including Logistic Regression (LR), Support Vector Machine (SVM), K Nearest Neighbor (KNN), Decision Tree (DT), Random Forest (RF), Gradient Boost Classifier (GBC), Multilayer Perceptron (MLP), and 1D Convolutional Neural Network (1DCNN) were developed and evaluated. Although the performance differences among the models were subtle (within a range of 0.001), the SVM classifier outperformed the others, achieving a recall of 0.9979 and an AUC of 0.9998. Consequently, the SVM model was selected for deployment to monitor and detect patients’ medication adherence levels, enabling timely interventions to improve patient outcomes.

**Conclusions:**

This study highlights a variety of machine learning (ML) models that can be effectively used to monitor and classify medication adherence in diabetic patients in Ethiopia. However, to fully realize the potential impact of digital health applications, further studies that include patients from diverse settings are necessary. Such research could enhance the generalizability of these models and provide insights into the broader applicability of digital tools for improving medication adherence and patient outcomes in varying healthcare contexts.

## Introduction

Non-adherence to medication significantly affects patient health outcomes and increases healthcare costs, particularly for those with diabetes, a prevalent chronic condition (1–4). Non-adherence with medications affects glycemic control, which in turn contributes to a significant proportion of hospitalizations, deaths, and expenditures related to drugs on higher healthcare system costs (5–11). Given that diabetes is a major public health threat expected to affect 783 million people by 2045, ensuring medication adherence is crucial (12).

Persistent long-term medication adherence is crucial for effective disease management in patients with diabetes. However, evidence indicates that a significant proportion of diabetes patients fail to adhere to their prescribed medications. In developed nations, up to 50% of patients report low adherence to long-term medications, and this issue is even more pronounced in low- and middle-income countries, where factors such as limited access to healthcare, financial constraints, and cultural barriers further exacerbate the problem. Addressing this challenge is essential to improving patient outcomes globally (13).

Studies worldwide have shown that medication nonadherence in patients with diabetes ranges from 6.3% to 87% (14–18). Similarly, studies conducted in Ethiopia also showed that the rate of drug nonadherence ranged from 41.5-76.9% (5, 19–26). The pooled prevalence of nonadherence to medications has also been reported to be high in meta-analysis studies (27, 28). These findings highlight the challenges associated with medication adherence and suggest that implementing an alarm system to monitor medication adherence could be a valuable strategy. Traditionally, predicting medication adherence has relied on static and group-based factors, such as medication tolerability, diagnosis, duration of treatment, and demographic information (29, 30). However, adherence can be influenced by multiple factors, including logistical issues such as forgetfulness, complex medication regimens, complications with prescription refills, side effects, adverse effects, lack of insurance coverage, and limited financial resources (5, 31, 32).

Assessing medication adherence is crucial for ensuring optimal patient outcomes, yet directly identifying adherence levels can be challenging for healthcare providers. Physicians often rely on methods such as self-reporting or pill counts, which may not accurately reflect a patient’s true adherence. This gap highlights the need for more reliable methods to detect nonadherence and take appropriate action. Achieving a precise and cost-effective assessment remains a significant challenge. However, leveraging assistive technologies, particularly machine learning (ML) models, could enhance early detection of nonadherence. By analyzing data from various sources, these models can provide healthcare providers with actionable insights, helping them allocate resources more effectively and improve patient care (33).

Research demonstrates that health data can provide ML models with valuable information for individual healthcare evaluation and analysis (34). ML models have proven especially useful for data analysis, prediction, and the detection of chronic conditions and related complications such as diabetes (35–38). In the context of chronic diseases, adherence is often measured using statistical methods (39). However, with the growing volume of healthcare data, predictive models based on ML techniques are increasingly utilized. Compared to traditional statistical methods, ML approaches offer distinct advantages, including the ability to capture nonlinear relationships, reduced bias through automated learning, and greater flexibility in preventing overfitting (40, 41).

Unlike traditional methods, which rely on predefined instructions, ML models are trained using real-world data. These models learn to map features to outcomes through algorithms, enabling them to generalize knowledge and make accurate predictions for new, unseen inputs (42). This paradigm shift toward ML applications has transformed chronic disease management, introducing personalized, data-driven precision care in place of traditional strategies.

Despite the increasing significance of machine learning (ML) in healthcare, our comprehensive literature review found no documented evidence of its application in assessing, monitoring, or collecting data on medication adherence among individuals with diabetes in the Ethiopian population. To address this gap, the present study developed and evaluated ML models designed to classify, monitor, and record medication adherence levels in patients with type 2 diabetes in Ethiopia. These findings contribute to the growing body of knowledge on technological solutions for predicting medication adherence, particularly within the Ethiopian context. Additionally, they provide a foundation for future research, offering insights into effective ML-based methods for improving patient outcomes and advancing healthcare practices in Ethiopia.

## Methods

### Study design, setting, and participants

A cross-sectional study design was used to collect data on medication adherence among patients with type 2 diabetes who attended the chronic care follow-up at the University of Gondar Comprehensive Specialized Hospital (UoGCSH) between February and May 2023. The study included adults (aged ≥ 18 years) who had been receiving diabetes medications for at least 3 months and were able to respond to the interview. The three months were chosen because it provides a sufficient time frame to assess medication adherence reliably, as it captures habitual patterns and allows treatment regimens for chronic conditions like diabetes to stabilize. This duration is commonly used in clinical studies and guidelines, offering a balance between obtaining meaningful data and enabling timely interventions. Exclusion criteria included patients with severe or acute illnesses requiring emergency treatment, individuals with severe neurological or psychiatric conditions who could not communicate effectively, pregnant women, and patients with incomplete data. This approach ensured that the study focused on individuals who could provide reliable information about their medication adherence.

The sample size was determined using the single mean proportion formula: n = p(1-p) Z²/d², with the following assumptions: a predicted response distribution for medication adherence using ML (P = 0.5), a 95% confidence interval, and a 5% margin of error (d = 0.05), yielding a sample size of 385. Considering a 10% non-response rate, the final study enrolled 422 participants. Using the medical record list as a sampling frame, participants were selected through simple random sampling and a lottery method. Signed informed consent was obtained from all participants, indicating their agreement to allow the use of their questionnaire responses and medical records for research purposes. Of the 422 participants, 13 were excluded due to their inability to respond to the interview, and 6 were excluded due to missing data in the medication adherence measurement items. Ultimately, 403 data entities were included in the final analysis.

### Model development procedure

**Figure 1:**
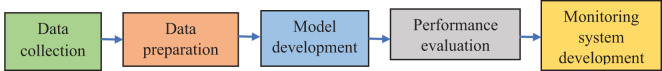
Study procedures for ML model development.

The development of the ML model was continued using data on patient medication adherence as determinant features of the model development that can distinguish medication adherence from patients with high and low levels of medication adherence. These determinant features were subsequently used to train an ML model. **Figure 1** shows the overall methodology used in this study.

### Instruments and data collection procedures

Demographic and medication-related data from the patients were collected using a structured interview-based questionnaire. Before the actual data collection, the data collection instrument was validated and ensured for its content and clarity. Then 4.5% of the study subjects in the study area were pretested (excluded from the final analysis) to ensure the completeness and consistency of the data collection tool. Then, an appropriate amendment was employed. The data were collected by experienced nurses and pharmacists after they had trained for two days. The supervisor explicitly clarified the purpose of the study, and the data collection tools and techniques. The data collection procedure was closely monitored. After the medical records were entered into Microsoft Excel 2013 and checked for repetition, the patients were interviewed, and the data were simultaneously extracted.

Medication adherence: in this study, it refers to the extent to which a patient actively, voluntarily, and consistently follows a mutually agreed-upon treatment plan, including taking medications as prescribed, in collaboration with their healthcare provider. It involves the patient’s commitment to take their medication by prescription that aims to achieve the desired therapeutic outcomes.

Medication adherence data were utilized as a key determinant in developing the ML model. Low and high levels of medication adherence were assessed using the General Medication Adherence Scale (GMAS), an 11-item interview-based questionnaire combining subjective and objective measures. Each item was rated on a 4-point Likert scale, ranging from 0 (lowest) to 3 (highest). The items were categorized into three factors: (I) patient behavior (5 items), (II) medication pill or injection burden (4 items), and (III) medication cost and payment (2 items). The 11 items for which responses were collected addressed various challenges related to medication adherence. These included difficulty remembering to take medications, forgetting medications due to busy schedules, travel, or other events, discontinuing medications when feeling well, stopping medications due to adverse effects, discontinuing medications without consulting a doctor, ceasing medications when prescribed additional treatments for other conditions, finding it burdensome to remember medications due to regimen complexity, missing doses because of disease progression or the addition of new medicines, altering medication regimen, dose, or frequency, discontinuing medications because they are perceived as not worth the cost, and difficulty purchasing medications due to high expenses. The responses to these 11 items, which determined the level of medication adherence as either low or high, were subsequently used to train and develop the ML model.

The English version of the GMAS has been validated (43) and the item has been previously applied to assess medication adherence within the Ethiopian population with a Cronbach’s alpha of 0.84, and its item-level content validity index exceeded 0.79 (5). Scores for each item were summed to calculate a total adherence score, which was used to classify patients’ adherence levels. A GMAS score of <26 indicated low adherence, while a score of ≥27 (out of a maximum of 33 points) was classified as high adherence (5, 44).

During data collection, most participants were found to have low adherence to their medications, with only 22.6% exhibiting a history of high medication adherence. This imbalance in class distribution could significantly affect the performance of machine learning models, as the model may become biased toward the majority class. To address this issue, the Synthetic Minority Oversampling Technique (SMOTE) was employed. SMOTE works by selecting instances from the minority class, identifying their nearest neighbors, and generating synthetic samples through interpolation between the selected instances and their neighbors. These synthetic samples are added to the dataset, effectively balancing the class distribution, improving the model’s performance, and reducing bias toward the majority class.

Additionally, class imbalance can cause issues when the dataset is split into training and testing subsets, where one class may dominate the training set while the other predominates in the test set. This could result in skewed outcomes and variability with each train-test split. To mitigate this, the stratified K-fold cross-validation technique was applied, ensuring that each fold maintains a proportional distribution of both classes, leading to more stable and reliable model evaluation.

### Classification models and performance evaluation

Once the data was read then it was subsequently divided into training and testing sets. Then training was performed using LR, RF, SVM, DT, KNN, and XGBC algorithms. The performance of each classification model was evaluated using the parameters precession, recall, F1 score, accuracy, and the area under the curve (AUC). The performance of a machine learning classifier can be measured using several performance evaluation metrics. In this study accuracy precision, recall, and f1-score are used to measure the performance of the developed models. Accuracy is the measure of the overall correctness of the model given by the formula:


$$accuracy=\frac{TP+TN}{TP+TN+FP+FN}$$

Where: TP (True Positives): The number of positive samples correctly identified by the model. TN (True Negatives): The number of negative samples correctly identified by the model. FP (False Positives): The number of negative samples incorrectly identified as positive. FN (False Negatives): The number of positive samples incorrectly identified as negative. Precision is the ratio of correctively classified positive observation to the total predicted positives and is given by the formula:


$$precision=\frac{TP}{TP+FP}$$

High precision indicates a low false positive rate. Recall is the ratio of correctly predicted positive values to all actual positive values given by the formula:


$$recall=\frac{TP}{TP+FN}$$

High recall indicates a low false negative rate. Recall is also called Sensitivity or True Positive Rate (45). False positive rate (FPR) is the ratio of actual negatives that are incorrectly classified as positive and all actual negatives given by the formula:


$$FPR=\frac{FP}{FP+TN}$$

F1 score is the harmonic mean of precision and recall given by the formula:


$$\;f1\;score\;=2*\frac{precision*recall}{precision+recall}$$

A binary classification model’s performance is graphically represented by the Receiver Operating Characteristic curve, or AUC-ROC curve that shows the degree or measure of separability. At different categorization criteria, it shows the true positive rate (45) against the false positive rate (FPR). As it gives a single scalar value ranging from 0 to 1 the total performance of the model is shown by the AUC_ROC score. ROC_AUC score can be obtained by the formula:


$$\text{AUC}-\text{ROC}=\int_{0}^{1}TPR\left(FNR\right)\text{d}FNR$$

For the perfect model, the AUC_ROC score will be 1, models that are not better than random guessing will score 0.5, and models worse than random guessing will score less than 0.5.

Extensive parameter tuning was done using grid search, random search, and optuna parameter tuning algorithms, and the best combination of hyperparameters was selected.

### Monitoring system development

Finally, the selected best performer model was deployed using a flask environment aiming to design a data collector, monitor, and classifier platform for medication adherence level.

## Results

### Data collection results

Out of 422 participants approached, data on medication adherence, used as a feature for machine learning, were analyzed from 403 patients, resulting in a 95.5% response rate. Most of the study participants (77.45%; 95% CI: 70.1-83.8) were found to have low medication adherence.

### Classification models and performance evaluation

**Figure 2:**
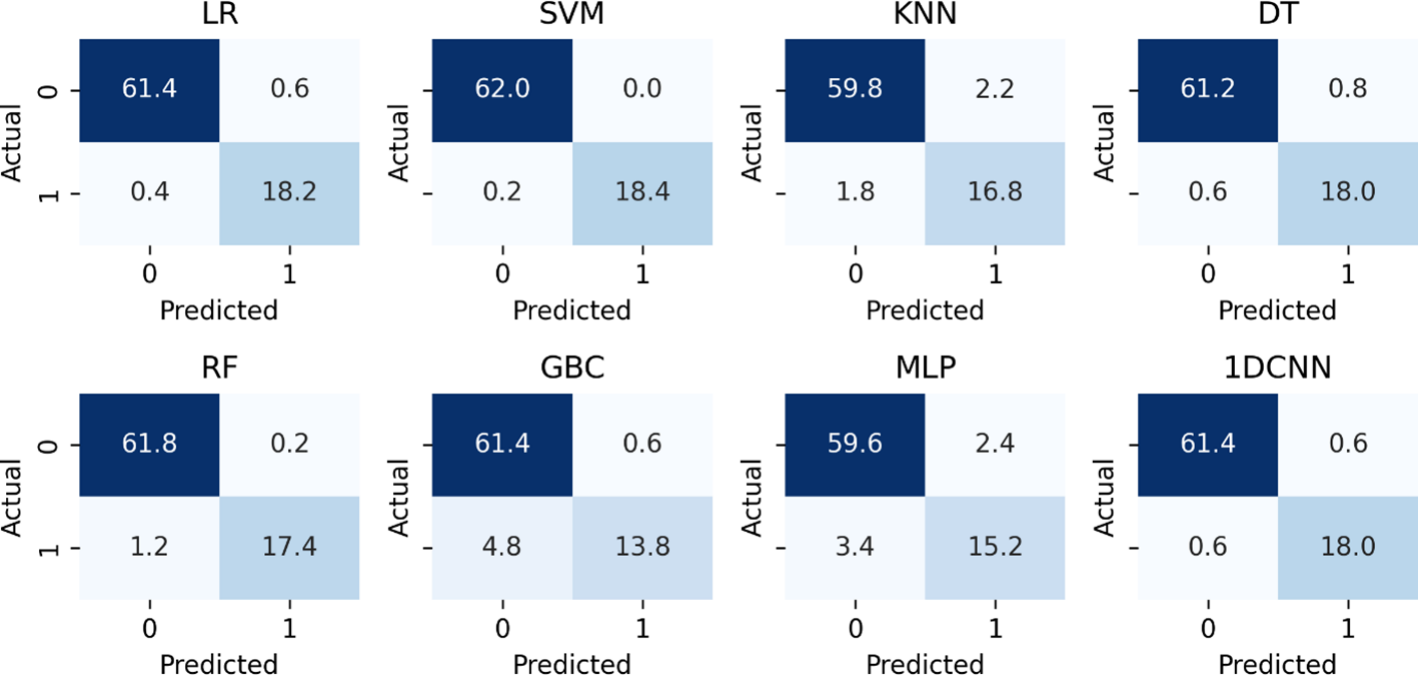
The confusion matrix scores of the developed models before balancing the data classes and tuning model parameters.

Using the extracted data features, ML models were developed using six ML algorithms. The performance of each model developed was evaluated. **Table 1** displays the model’s accuracy, AUC-ROC score, recall, precision, and F1 score before the application of SMOTE. SVM performs better with the values 0.9948 and 1.00 for recall and AUC_ROC, respectively. From the confusion matrix plots before SMOTE shown in **Figure 2**, we can see that SVM misclassifies an average of 0.2 out of 80.6 entities, which is the lowest value.

**Table 1:** The accuracy, precision, recall, and F1 score of the developed models before balancing the data classes and tuning model parameters.

Models	Performance evaluation report				
	Accuracy	Precision	Recall	F1-score	AUCROC
Logistic Regression	0.9876	0.9809	0.9731	0.9684	1.0000
Support Vector Classifier	0.9975	0.9900	0.9948	1.0000	1.0000
K Neighbors Classifier	0.9504	0.9114	0.8938	0.8852	0.9947
Decision Tree Classifier	0.9825	0.9675	0.9601	0.9575	0.9774
Random Forest Classifier	0.9827	0.9375	0.9589	0.9875	0.9998
Gradient Boosting Classifier	0.9330	0.7497	0.8312	0.9666	0.9866
Multilayer Perceptron Classifier	0.9277	0.7775	0.7332	0.6975	0.8726
1D convolutional neural network	0.9875	0.9804	0.9744	0.9701	0.9998

The goal of the model is to classify medication adherence levels. This is somewhat acceptable if the model categorizes low non-adherence levels as high. However, suppose the model incorrectly identifies high non-adherence levels as low. In that case, it can be critically harmful, as it would mislead both the patient and the physician, potentially delaying necessary corrective measures. A high recall value indicates a low false negative rate, meaning that the number of instances where high non-adherence levels are incorrectly predicted as low non-adherence levels are minimal. Therefore, models that demonstrate higher accuracy and recall are considered better. Among the eight models evaluated, three performed better in accuracy and recall. The collected data is highly imbalanced, with 93 instances (22.6%) of high non-adherence and 310 instances (77.4%) of low non-adherence, which significantly unbalanced data for machine learning models. To address this issue, the Synthetic Minority Over-sampling Technique (SMOTE) was used. The balanced data’s class distribution is 50% for the low adherence class and 50% for the high adherence class detailed in **Table 2**.

**Table 2:** The amount of data in each class before and after applying the SMOTE technique.

Applying smote	Number of outputs at each level	
	Low adherence	high adherence
Without	310	93
With	310	310

**Figure 3:**
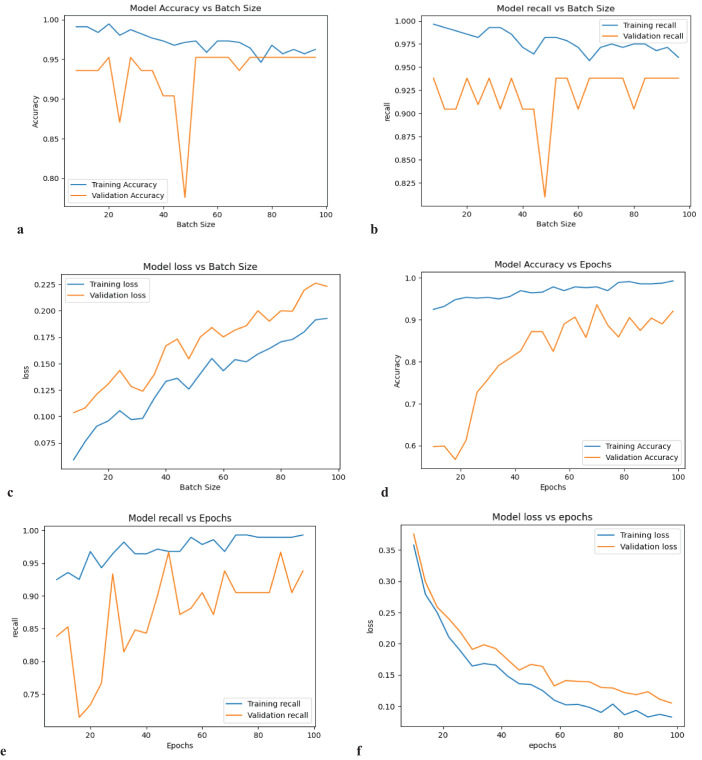
The performance of the 1DCNN model while tuning parameters **(a)** accuracy vs batch size, **(b)** recall vs batch size, **(c)** loss vs batch size, **(d)** accuracy vs epoch, **(e)** recall vs epoch and **(f)** loss vs epoch.

For the first seven machine learning models the parameters are not bulky and parameter tuning was done manually. Again, for the case of 1DCNN, it was computationally inefficient to tune all 1DCNN parameters automatically; as a result, the number of epochs and the batch size were tuned manually, and the performance of the model with the respective batch size and epochs is shown in **Figure 3**.

**Figure 4:**
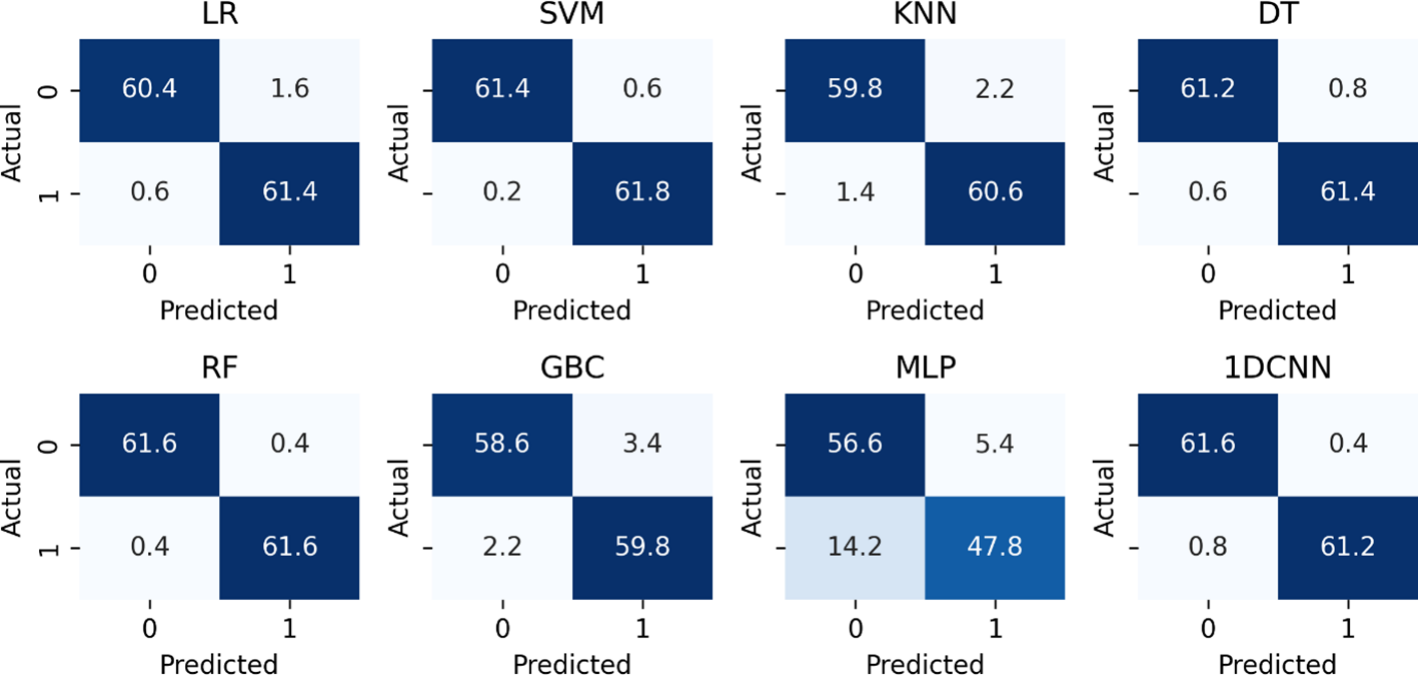
The confusion matrix score of each model after applying SMOTE and parameter tuning.

Considering the computation time, recall score, accuracy, and loss, batch size 20 is the optimum value. Again, by considering the computation time, recall score, accuracy, and loss, the optimum number of epochs is 90. Finally, after applying SMOTE to the data and tuning parameters, the SVM model is performing superior to others, with a recall of 0.9969 and an AUC score of 0.9998, as presented in **Table 3**. Furthermore, **Figure 4**’s confusion matrix computation reveals an average misclassification of 0.8 for the SVM model among 124 entities.

**Table 3:** The accuracy, precision, recall, F1 score, and AUC_ROC scores of the developed models after data class balancing and parameter tuning.

Models	Performance evaluation report				
	Accuracy	Precision	Recall	F1-score	AUC_ROC
Logistic Regression	0.9822	0.9739	0.9898	0.98183	0.9992
Support Vector Classifier	0.9935	0.9903	0.9969	0.99357	0.9998
K Neighbors Classifier	0.9709	0.9635	0.9785	0.97036	0.9981
Decision Tree Classifier	0.9903	0.9902	0.9907	0.99042	0.9906
Random Forest Classifier	0.9951	0.9938	0.9962	0.99502	0.9998
Gradient Boosting Classifier	0.9596	0.9543	0.9637	0.95895	0.9805
Multilayer Perceptron Classifier	0.8419	0.7387	0.7838	0.7579	0.8484
1D convolutional neural network	0.9951	0.9968	0.9932	0.9949	0.9996

**Figure 5:**
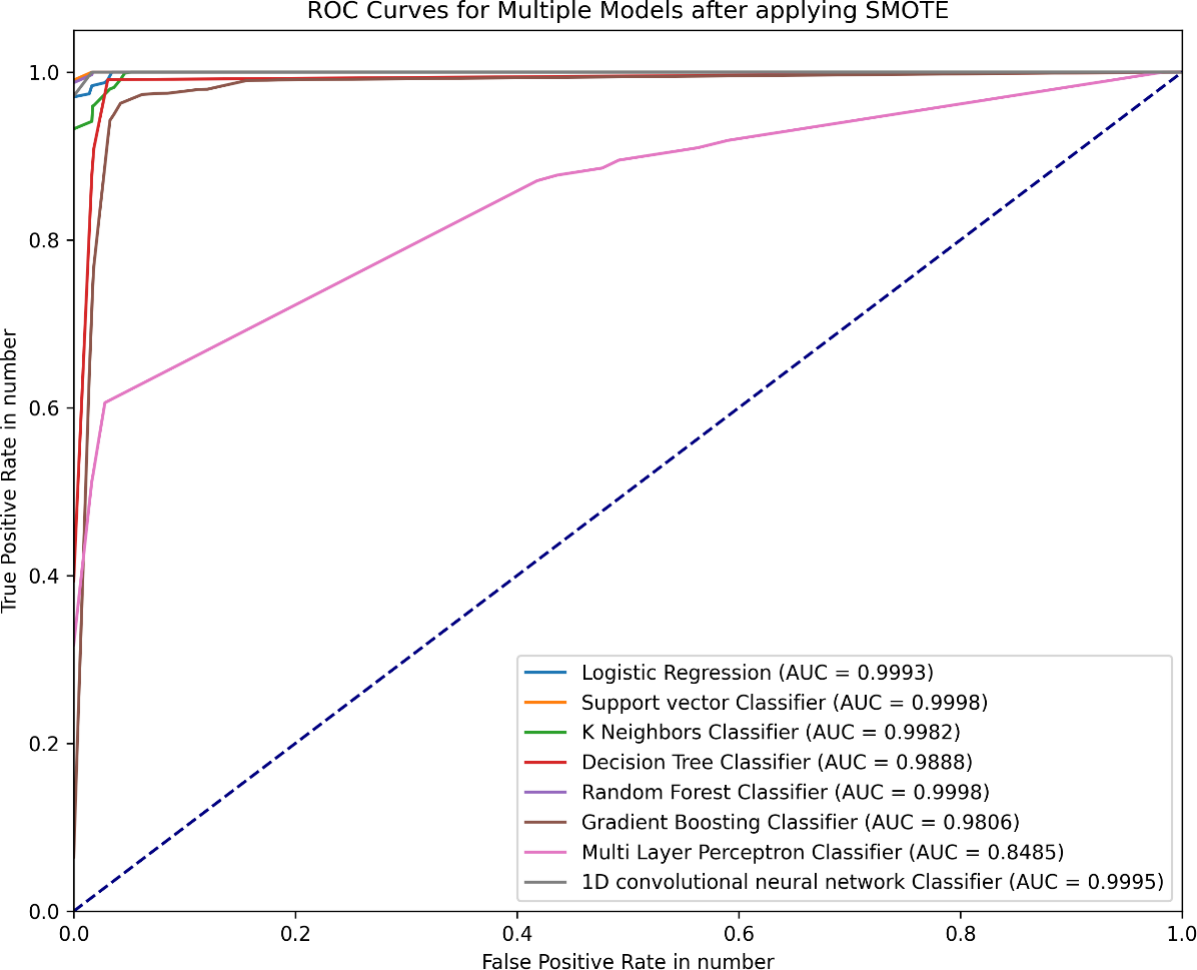
The ROC curve of the optimized models.

**Figure 5** shows the region of convergence (ROC) graphs of the final optimized models.

### Monitoring system development

**Figure 6:**
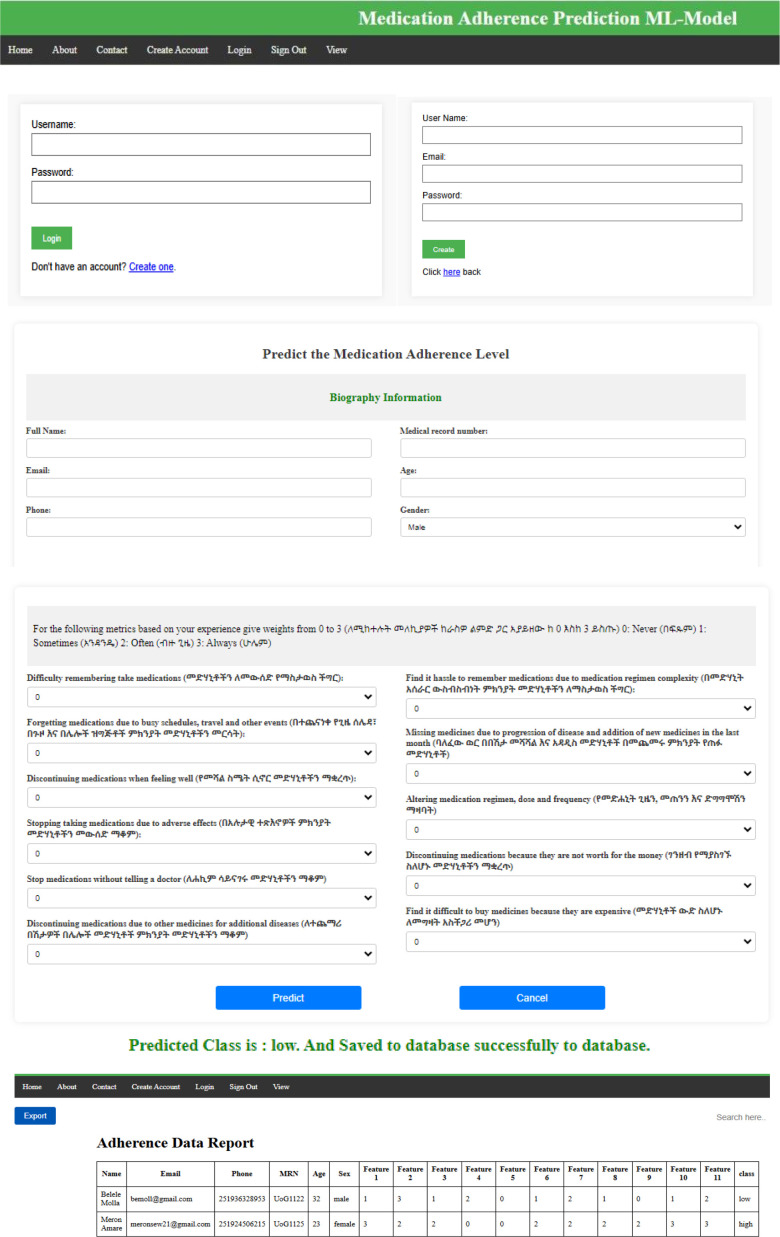
The overview of the developed monitoring system, the home page, the login page, the demographic data entry page, the GMAS data entry page, and the monitoring window page.

The Support Vector Classifier model was deployed on a flask environment as a web-based application. **Figure 6** shows an overview of the web-based applications. The deployed application enables the subjects with diabetes to fill out the GMAS parameters and assess their adherence level, the physicians to monitor the patient’s medication adherence level, and the data scientist to access the collected medication adherence data from the database.

## Discussion

Diabetes is one of the biggest health risks worldwide, expected to affect 783 million people by 2045 (5–8, 11). In developing countries like Ethiopia, poor medication adherence has become a significant contributor to mortality, hospitalization, and financial strain (13, 19–22, 25, 26). ML algorithms offer a promising solution to this issue and have shown encouraging results. However, models developed in one country may not be applicable to others due to differences in data. This study aimed to develop and implement a machine learning (ML) model to classify and monitor medication adherence levels, as well as to gather data on medication adherence. Data on medication adherence among diabetic patients were collected using the GMAS questionnaire tool [5].

With promising recall (true positive rates) of 0.9898, 0.9969, 0.9785, 0.9907, 0.9962, 0.9637, 0.7838, and 0.9932, and AUC-ROC scores of 0.9992, 0.9998, 0.9981, 0.9906, 0.9998, 0.9805, 0.8484, and 0.9996 for the LR, SVM, KNN, DT, RF, GBC, MLP, and 1DCNN models, respectively, the study’s findings demonstrated that machine learning models can effectively classify medication adherence. Although all the models except the multilayer perceptron model performed very well, the support vector classifier model achieved the best recall and AUC scores before and after addressing the data class imbalance and performing significant parameter adjustments. This study discusses the findings using other international studies conducted on patients with diabetes and other chronic diseases because of a lack of similar studies in a local context. Consistent with the current study, a study used 18 machine learning models for predicting medication adherence of diabetic patients obtained an AUC score of 0.716, 0.743, 0.698, 0.672, 0.667, 0.717, and 790 for LR, RF, SVM, DT, KNN, XGBC, and ensemble models respectively (46). A study on predicting medication adherence levels in people with type 2 diabetes found that the KNN model had an AUC of 0.838 and the SVM model had an AUC of 0.765 (47). Another study that employed logistic regression, multilayer perceptron, and convolutional neural networks to predict medication adherence from Continuous Glucose Monitoring (CGM) signals resulted in accuracy scores of 0.652, 0.725, and 0.775 (48). While most of the findings are in line, little discrepancies between the current study and these previous studies could be due to variations in data collection and analysis methods, with some studies employing in-person questionnaires and electronic medical records. However, the ML models developed in the current study performed remarkably well. This study achieved a better AUC_ROC score, suggesting that its implementation will contribute significantly to monitoring medication non-adherence.

A study on opioid medication adherence classification using LR, DT, RF, and XGBC models reported accuracy scores of 0.9415, 0.8787, 0.9411, and 0.9417, respectively (49). These results indicate that such models are well-suited for implementation in healthcare settings. Similarly, another study on medication adherence in Crohn’s disease patients showed average classification accuracies of 0.816, 0.859, and 0.877 for LR, Backpropagation Neural Network, and SVM models, respectively (40). This suggests that the current study has achieved strong comparative results. Discrepancies between studies may stem from differences in adherence measurement techniques, the nature of the data, and population characteristics.

In another example, Artificial Neural Networks, RF, and Support Vector Regression models achieved accuracies of 0.65, 0.78, and 0.79, respectively, in predicting medication adherence (50). Additionally, a study on predicting medication adherence reported accuracies of 0.777 and 0.772 for XGBC and RF models, respectively (51). Another study using XGBC to classify medication adherence achieved an accuracy of 0.722 in the early stages of research (52). The application of SVM models for medication adherence classification achieved an accuracy of 0.776 using data from 76 patients with heart failure (53). These results align with the findings of this study, supporting the idea that the models developed are suitable for implementation in real-world healthcare settings. Furthermore, a study predicting medication adherence levels in hypertension patients reported AUC scores of 0.774, 0.914, and 0.866 for LR, DT, and RF models, respectively (54). The stronger performance of our study further underscores its potential for confident implementation in healthcare facilities.

A study that developed 300 prediction models using 30 machine ML algorithms demonstrated that medication adherence in patients with type 2 diabetes was predicted with greater precision as the volume of input data increased (54). While further research is needed to assess the potential of ML-based techniques for measuring adherence in patients with chronic infectious diseases, these methods have shown promise for evaluating medication adherence in patients with noncommunicable diseases (3, 33, 55).

### Strengths and limitations of this study

This study lays a critical foundation for leveraging technology to improve healthcare and has significant potential for scalability. The innovative use of machine learning to develop a medication adherence system could revolutionize diabetes management in Ethiopia, with the ability to enhance patient outcomes and inform public health strategies. Moreover, the collected data on medication adherence provides valuable insights that can be used to improve patient care, shape public health policies, and guide future research.

However, it is important to note that the data used in this study do not represent the national population, and the findings should be interpreted with caution. Additionally, medication adherence was assessed using a self-reported tool that combines both subjective and objective measures, which may impact the overall adherence outcome. Self-reported data can sometimes introduce biases, such as overreporting or underreporting of adherence, which must be considered when evaluating the results. Furthermore, while machine learning algorithms provide valuable insights, all decisions are supervised by human experts, ensuring that the results are interpreted and applied responsibly. Therefore, the findings should be approached with caution, and future research should focus on expanding the sample to better represent the national population and exploring additional data collection methods to minimize biases.

### Implications for research, practice, and policy

#### Research implications

This study provides a foundation for future research on the application of machine learning (ML) in healthcare, specifically in monitoring medication adherence in patients with type 2 diabetes. Future research should aim to include larger, more diverse patient populations to better assess the generalizability of ML models across various demographics, settings, and healthcare environments. Additionally, exploring the integration of digital tools, such as mobile applications and wearable devices, with ML models could further enhance their ability to track and predict medication adherence patterns, leading to improved patient outcomes.

#### Practice implications

In clinical practice, implementing ML models, such as the support vector machine used in this study, offers an innovative approach to accurately monitor medication adherence in patients with diabetes. Healthcare providers can leverage these technologies to identify non-adherence early, personalize treatment plans, and intervene promptly to improve patient compliance. Furthermore, the automated monitoring and data collection systems can reduce clinician workload, streamline the management of chronic conditions, and enhance the overall efficiency of diabetes care.

#### Policy implications

The findings highlight the potential of ML-driven solutions to improve medication adherence, which could be integrated into national or regional healthcare policies for managing chronic diseases like diabetes. Policymakers should promote the inclusion of digital health tools and ML models into routine clinical practices, particularly in low-resource settings such as Ethiopia. Investments in healthcare infrastructure that supports digital applications could address barriers to medication adherence, reduce healthcare costs, and improve public health outcomes. To ensure equitable access to these technologies, policymakers should also focus on addressing potential barriers such as data privacy concerns, training for healthcare professionals, and infrastructure limitations in underserved regions.

## Conclusion

While all the developed models performed well in classifying medication adherence levels in patients with type 2 diabetes, the SVM model outperformed the others based on its recall and AUC scores, both before and after applying the SMOTE data balancing method. This suggests that SVM may be the most effective model for predicting medication adherence in this context. Therefore, ML models, particularly SVM, should be further investigated and implemented in the Ethiopian healthcare system to optimize medication adherence for patients with chronic diseases like diabetes. However, nationally representative data, including diverse patient populations from various regions of the country, is essential to better validate the role of these technology-assisted models and ensure their applicability across different healthcare settings. Future research should also explore practical considerations for integrating these models into routine clinical practice, including infrastructure, training for healthcare providers, and policies that support digital health solutions.

## Data Availability

The original contributions presented in the study are included in the article/supplementary material. Further inquiries can be directed to the corresponding author.
